# An SPRi Biosensor for Determination of the Ovarian Cancer Marker HE4 in Human Plasma

**DOI:** 10.3390/s21103567

**Published:** 2021-05-20

**Authors:** Beata Szymanska, Zenon Lukaszewski, Beata Zelazowska-Rutkowska, Kinga Hermanowicz-Szamatowicz, Ewa Gorodkiewicz

**Affiliations:** 1 Bioanalysis Laboratory, Faculty of Chemistry, University of Bialystok, Ciolkowskiego 1K, 15-245 Bialystok, Poland; b.szymanska@uwb.edu.pl; 2 Faculty of Chemical Technology, Poznan University of Technology, pl. Sklodowskiej-Curie 5, 60-965 Poznan, Poland; zenon.lukaszewski@put.poznan.pl; 3 Department of Pediatric Laboratory Diagnostics, Medical University of Bialystok, Waszyngtona 17, 15-274 Bialystok, Poland; zelazowskab@wp.pl; 4 Comprehensive Cancer Center, Department of Clinical Oncology, Ogrodowa 12, 15-027 Bialystok, Poland; kiniaziel@wp.pl

**Keywords:** cancer markers, ovarian cancer, HE4, SPRI biosensors, array of measuring points

## Abstract

Human epididymis protein 4 (HE4) is an ovarian cancer marker. Various cut-off values of the marker in blood are recommended, depending on the method used for its determination. An alternative biosensor for HE4 determination in blood plasma has been developed. It consists of rabbit polyclonal antibody against HE4, covalently attached to a gold chip via cysteamine linker. The biosensor is used with the non-fluidic array SPRi technique. The linear range of the analytical signal response was found to be 2–120 pM, and the biosensor can be used for the determination of the HE4 marker in the plasma of both healthy subjects and ovarian cancer patients after suitable dilution with a PBS buffer. Precision (6–10%) and recovery (101.8–103.5%) were found to be acceptable, and the LOD was equal to 2 pM. The biosensor was validated by the parallel determination of a series of plasma samples from ovarian cancer patients using the Elecsys HE4 test and the developed biosensor, with a good agreement of the results (a Pearson coefficient of 0.989). An example of the diagnostic application of the developed biosensor is given—the influence of ovarian tumor resection on the level of HE4 in blood serum.

## 1. Introduction

Human epididymis protein 4 (HE4), also known as recombinant WAP four disulfide core domain protein 2 (WFDC2), is used as an ovarian cancer marker. HE4 may be used as a marker of ovarian cancer alone or jointly with the other ovarian cancer marker CA 125 as a component of ROMA (risk of ovarian malignancy algorithm) [1,2]. The marker may be present in several isoforms with different molecular weights [1,3]; therefore, its concentration is usually expressed in pM (picomole/L). Serous HE4 concentration is strongly elevated in the case of ovarian cancer, although a certain elevation is also observed in cervical and breast cancers [4], as well as in chronic kidney disease [5]. Moreover, marker concentration strongly depends on the stage of ovarian cancer. The HE4 concentration above which ovarian cancer is diagnosed (cut-off) depends on menopausal status, and surprisingly, the method used for HE4 determination [6,7]. Four analytical methods are currently used for serum HE4 diagnostics: ELISA, chemiluminescence enzyme immunoassay (CLEIA), electrochemiluminescence immunoassay (ECLIA), and chemiluminescent microparticle immunoassay (CMIA) [1]. These methods are implemented using commercially available automated immunoassays provided by Fujirebio Diagnostics (ELISA and CLEIA), Abbott Diagnostics (CMIA), and Roche Diagnostics (ECLIA) with various cut-off values. Despite the different detection techniques, these four immunoassays all have a sandwich construction consisting of two antibodies. The first antibody is used for HE4 entrapment from the analyzed sample, while the second is conjugated with a label that creates the analytical signal [6,7]. In the case of the most frequently used ECLIA, the second antibody is conjugated with a ruthenium(II) bipyridyl complex, which generates a light impulse triggered by the potential impulse. The alternatively used HE4 ELISA kit [8] also contains a label creating an indirect colorimetric analytical signal. In attempts to develop new methods for HE4 determination, more attention should be paid to label-free analytical techniques such as electric cell-substrate impedance sensing (ECIS), quartz crystal microbalance (QCM), surface acoustic wave (SAW), and SPR. A simple localized surface plasmon resonance (LSPR) biosensor with silver nanoparticles and the anti-HE4 antibody was developed for marker determination in blood serum [8]. This biosensor was updated for analytical signal enhancement [9]. Protein G is covalently immobilized on the silver nanoparticles, and an anti-HE4 antibody is attached to protein G. Such a biosensor is suitable for the determination of the HE4 marker in urea. Recently, a molecularly-imprinted photo-electrochemical sensor was developed for HE4 determination [10], as was a chemiluminescence immunoassay with HE4 accumulation on magnetic particles [11]. The aim of the present paper was to develop a much simpler, label-free immunosensor for HE4 determination based on a single antibody and the SPRi technique.

**Figure 1:**
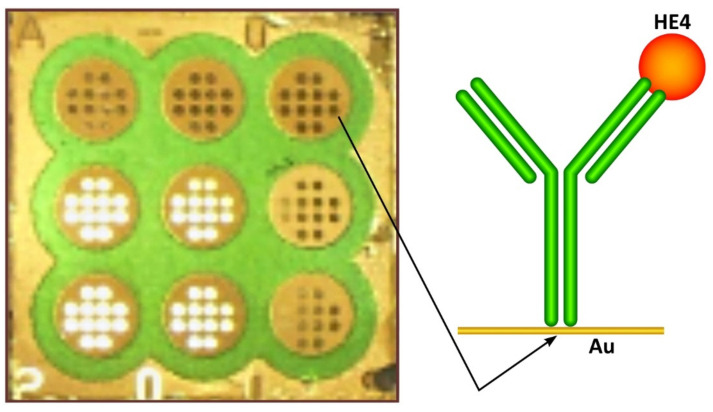
Chip architecture and sketch of biosensor.

The non-fluidic array version of the SPRi technique, already used for the determination of the ovarian marker CA 125 [12], exhibits analytical characteristics (LOQ) suitable for the determination of cancer markers in blood. In contrast to fluidic SPR, a biosensor is created ex situ, measurement is performed after the gentle removal of processing liquids [13], and no signal enhancements is needed to attain the required LOQ. An array of 9 × 12 measuring points enables the measurement of nine different samples [14] (see Figure 1). The chips are regenerable [15]. In the SPR imaging technique, a surface plasmon resonance signal is converted into an image recorded by a CCD camera. This differs from fluidic SPR, where a sensorgram is recorded, i.e., changes of an SPR signal in time, and from localized SPR, where the analytical signal is a shift in the localized surface plasmon resonance spectrum. Certainly, the physical nature of signal creation is the same in all three techniques: the successive immobilization of a receptor and an analyte causes changes in the refractive index on the chip surface and consequently changes in the SPR signal (a lowering of the SPR dip and a shift of the angle of polarized light). SPRi simply records reflected light using a CCD camera. The non-fluidic array version of the SPRi technique enables the determination of approximately 20 biomarkers without signal enhancement or the preliminary preconcentration of analyte [16,17], which is the advantage of this technique over the fluidic version of SPR.

Serous ovarian cancer samples exhibit an HE4 concentration above 150 pM, with maximal values above 850 pM [4], while the marker level for healthy premenstrual women ranges between 15 and 62 pM [18]. The cut-off value for HE4 in serum is recommended at between 70 and 87 pM for premenstrual and between 112 and 140 pM for postmenstrual women, depending on the method used for marker determination [6,7]. HE4 exhibits good selectivity and specificity (with an ROC (receiver operating characteristic) curve with an area under the curve (AUC) value of 0.91) and strongly reacts to the disease stage [4]. It should be noted that urine HE4 concentration is much higher than the serous marker concentration, and it is significantly elevated in the case of ovarian cancer [5]. The designed biosensor appears to be suitable for the determination of HE4 in both healthy individuals and ovarian cancer patients. Initial experiments showed that a biosensor consisting of cysteamine as the linker and covalently immobilized rabbit polyclonal antibody against HE4 is a suitable tool for the determination of the marker in blood plasma. Cysteamine (mercaptoethyleneamine) is bonded to the gold surface by a mercapto group, while an amine group enables the attachment of the antibody via the NHS/EDS protocol. The carboxy group of the Fc region of the antibody (opposite the Fab antigen-binding site) is used to form an amide bond with the amine group of cysteamine.

## 2. Experimental

### 2.1. Materials and Reagents

Human epididymis protein 4 (HE4) (MW of 44 kDa) (Cloud-Clone Corp., Houston, TX, USA), rabbit polyclonal antibody against HE4 (ABCAM plc, Cambridge, MA, USA), cysteamine hydrochloride, N-ethyl- N’-(3-dimethylaminopropyl) carbodiimide (EDC), human albumin (all SIGMA, Steinheim, Germany), and N-hydroxysuccinimide (NHS) (ALDRICH, Munich, Germany) were used. The bases of the biosensors were chips covered with a layer of gold (Sens, Netherlands).

A complete list of materials and reagents is given in Appendix A.1.

### 2.2. Biological Material

The plasma samples used for the tests came from donors from the Regional Blood Donation and Blood Treatment Center in Bialystok, as well as from patients with ovarian cancer and endometrial cyst from the Maria Sklodowska-Curie Oncology Center. All samples were provided after obtaining the consent of the Bioethical Commission.

### 2.3. Procedures

#### 2.3.1. Antibody Immobilization 

In the first stage of antibody immobilization, the gold chip was covered with cysteamine linker by immersion in a 20 nM ethanolic cysteamine solution for at least 12 h; the chip was subsequently rinsed with absolute ethanol and water, and it was dried under an argon stream. The chip with cysteamine was stable over several months. Antibody immobilization was performed following the EDS/NHS protocol, as described (for example) in [19], with rabbit polyclonal antibody against HE4. For this purpose, the antibody was activated with a mixture of EDC (250 nM) and NHS (250 nM) in a carbonate buffer (pH 8.5) and applied to the chip previously covered with cysteamine. After incubation for 60 min at 37 °C, the chip was washed with water. In order to eliminate nonspecific adsorption, after the incubation of the chip with the antibody, a bovine serum albumin (BSA) solution with a concentration of 1 ng mL^−1^ was applied to the biosensor active sites and then washed several times with redistilled water before being dried under a stream of argon.

#### 2.3.2. SPRi Measurements

SPRI measurements were performed as previously described [14,20]. A home-made apparatus and chip architecture were used, as described in Appendix A.2. The chip architecture and a sketch of the biosensor are shown in Figure 1. The SPRi signal was measured twice, after the immobilization of the antibody and then after interaction with a solution containing HE4 at a constant light angle. A 3 μL plasma sample or a standard solution of HE4 was placed on an immunosensor for 10 min and then washed with an HBS-ES buffer and water. The buffer and water were removed from the chip surface by means of a vacuum.

## 3. Results and Discussion

### 3.1. Optimization of Antibody Concentration

The optimization of the concentration of the rabbit polyclonal antibody against HE4 was performed at a constant HE4 concentration (114 pM). A description of these experiments is given in Appendix A.3. On the basis of these experiments, an antibody concentration of 20 ng mL^−1^ was selected as the optimal value for further experiments.

### 3.2. Dependence of the Analytical Signal on HE4 Concentration. Calibration Graph

**Figure 2:**
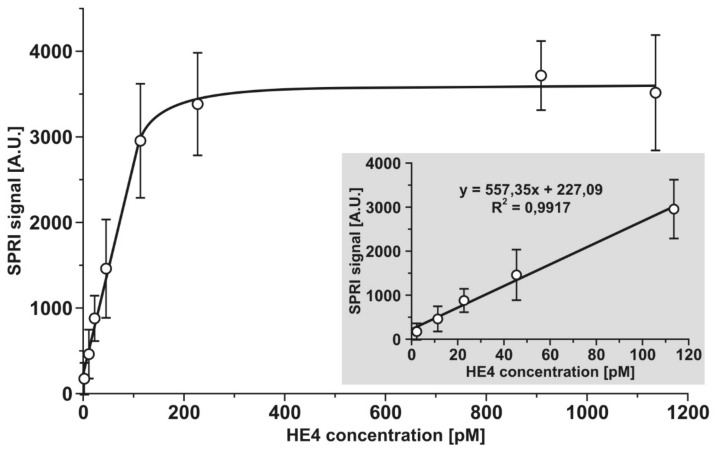
Dependence of the analytical signal on HE4 concentration. Calibration graph.

The analytical response of the developed biosensor was investigated for a range of HE4 concentrations between 2 and 1140 pM. Experiments were performed as described in Section 2.3.1 and Section 2.3.2 at an antibody concentration of 20 ng mL^−1^. The results are plotted in Figure 2. The typical Langmuirian shape of the curve was the result of the gradual saturation of the active points of the biosensor. The initial section of the curve was linear and suitable for analytical purposes. The linear range was from 2 to 110 pM. Higher concentrations could be determined after suitable dilution. Compared to the results of Yuan et al. [8], who also used an anti-HE4 antibody as a reception element and reported linearity in the semilogarithmic co-ordinate system from 10 pM, this work provided straight linearity from 2 pM. On the other hand, the biosensor described in this paper requires the dilution of more concentrated samples, while that of Yuan et al. was found to ensure linearity over three orders of magnitude of HE4 concentration [8].

### 3.3. Precision and Recovery. Limit of Detection 

Three series of experiments were performed to investigate the precision and recoveries of the developed biosensor. The selected spikes of HE4 are located at the beginning, middle, and end of the linear section of the calibration graph. Each series consisted of 24 single measurements. The obtained results are shown in Table 1.

The results showed that the precision and recoveries of the measurements of HE4 concentration made using the developed biosensor were acceptable and typical for such a determination. The limit of detection determined on the basis of three SD of blank was 2 pM.

### 3.4. Specificity of the Developed Biosensor

**Figure 3:**
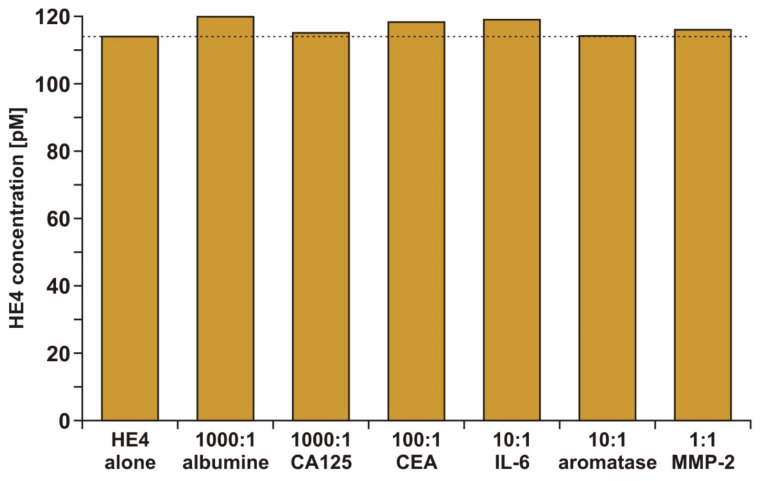
Specificity of HE4 determination. The determination of HE4 (114 pM) in the presence of variable excesses of potential interferents.

The specificity of the analytical response of the developed biosensor is a crucial factor, because in natural samples, the HE4 marker is present in an environment containing a huge number of other biologically active substances. Albumin and markers (cancer antigen 125 (CA 125), carcinoembryonic antigen (CEA), interleukin 6 (IL-6), aromatase, and metalloproteinase 2 (MMP 2)) were selected for these experiments. The highest excess of albumin and the CA 125 marker (1000:1) was applied because albumin is the most common protein in human blood and CA 125 is frequently used jointly with HE4 in ovarian cancer diagnosis. A constant HE4 concentration (114 pm), together with different excesses of potential interferents, was used in all tests. The results are shown in Figure 3.

The results showed that none of the investigated potential interferents had a significant influence on the HE4 analytical signal.

### 3.5. Validation of the Developed Biosensor

**Figure 4:**
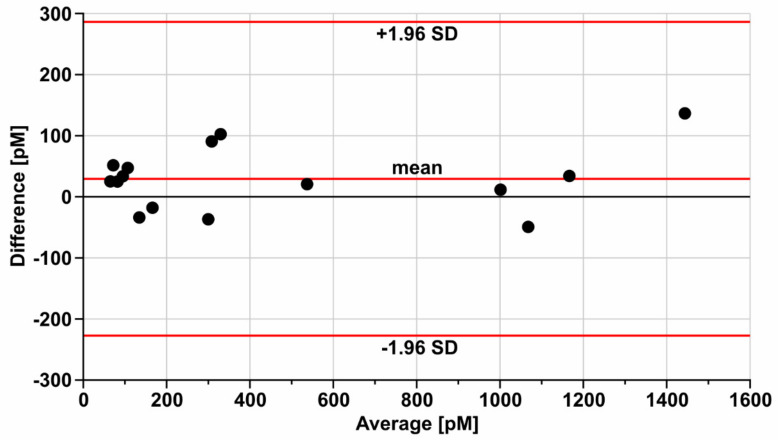
Bland-Altman plot for the HE4 results determined by a standard method (Elecsys HE4 performed on COBAS E-411) and the developed biosensor.

The validation of the developed biosensor was performed by the parallel determination of HE4 by the developed biosensor and a standard method in a series of blood serum samples from patients with ovarian cancer. The standard method was the Elecsys HE4 electrochemiluminescence performed on COBAS E-411. The results showed an acceptable correlation (a Pearson coefficient of 0.989). For a better comparison of the results of both methods, a Bland–Altman plot was made (Figure 4).

The plot indicated the acceptable agreement of both methods. The results obtained with the developed biosensor exceeded those of the standard method by approximately 20 pM. This difference was acceptable when considering that four currently used automatic methods for HE4 determination give different results [6,7]. Thus, the validation of the developed biosensor was completed.

### 3.6. Example of Diagnostic Application

**Figure 5:**
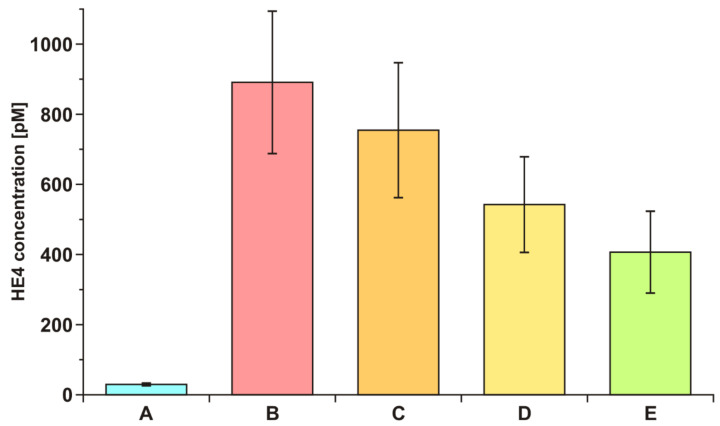
HE4 concentration in blood serum of ovarian cancer patients (n = 7) before (B) and after 6 h (C), 24 h (D), and 5 days (E) after tumor resection. (A) healthy volunteers (n = 18).

To demonstrate the diagnostic effectiveness of the developed biosensor, the HE4 concentration in the blood serum of ovarian cancer patients before and after tumor resection was investigated. Serum from seven patients was tested for HE4 concentration before resection and after 6 h, 24 h, and 5 days after resection. Average values are shown in Figure 5. The average value of HE4 concentration in blood serum is included for comparison.

A very fast decrease in the HE4 concentration after resection was observed, as was a much higher HE4 level in the serum of ovarian cancer patients than in healthy donors. These experiments confirmed that the developed biosensor may be a useful tool in the diagnosis of the disease.

## 4. Conclusions

A biosensor suitable for HE4 determination in the blood plasma of both healthy subjects and ovarian cancer patients was developed. The biosensor exhibited a linearity of the analytical signal response and acceptable precision and recovery. It was validated by the parallel determination of a series of plasma samples from ovarian cancer patients using the Elecsys HE4 test and the developed biosensor, with an acceptable agreement of the results. The influence of ovarian tumor resection on the level of HE4 in the blood serum, as determined by the developed biosensor, provided examples of its diagnostic application. The developed biosensor can extend the series of previously developed biosensors used with the non-fluidic array version of the SPRi technique, including biosensors for the determination of cathepsins B, D, G, L, and S; laminin-5; fibronectin; collagen IV; proteasome 20S and immunoproteasome 20S; UCHL-1; MMP-1 and MMP-2; aromatase; podoplanin; leptin; CA 125; CEA; and cystatin C. Many of these biosensors have been used in clinical investigations. Thus, a new diagnostic tool has been added to this series. Certainly, the simple construction of biosensors in the non-fluidic array version of the SPRi technique has some limitations. To resolve more complex analytical problems such as microRNA [21] and anti-cancer drug bleomycin [22] determination in human serum, an amplification of the SPR signal could be used.

## Figures and Tables

**Table 1:** Precision and recovery of HE4 with the developed biosensor (n = 24).

Added (pM)	Found (pM)	SD (pM)	RSD (%)	Recovery (%)
11.4	11.6	0.68	6.0	101.8
45.5	46.5	4.5	9.9	102.1
114	118	11	9.6	103.5

**Figure A1:**
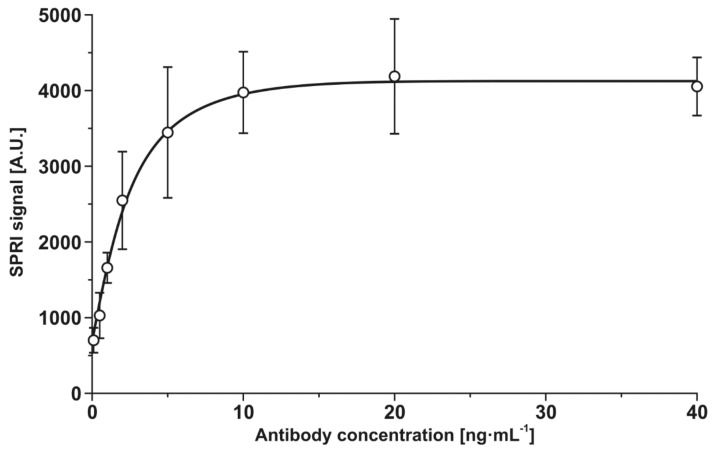
Dependence of the analytical signal on the concentration of rabbit polyclonal antibody against HE 4, at an HE 4 concentration of 114 pM.

## Data Availability

Not applicable.

## Appendix A

### Appendix A.1. Materials

Human epididymis protein 4 (HE 4) (M.W. 44 kDa)(Cloud-Clone Corp., USA), rabbit polyclonal antibody against HE 4 (ABCAM plc, Cambridge, USA), cysteamine hydrochloride, N-Ethyl- N’-(3-dimethylaminopropyl) carbodiimide (EDC), human albumine, (all SIGMA, Steinheim, Germany) and N-Hydroxysuccinimide (NHS) (ALDRICH, Munich, Germany) were used, as well as absolute ethanol (POCh, Gliwice, Poland), HBS- ES solution pH = 7.4 (0.01 M HEPES, 0.15 M sodium chloride, 0.005% Tween 20, 3 mM EDTA), carbonate buffer pH = 8.50–9.86, Phosphate Buffered Saline (PBS) pH = 7.4, (all BIOMED, Lublin, Poland), HBS- ES solution pH = 7.4 (0.01 M HEPES, 0.15 M sodium chloride, 0.005% Tween 20, 3 mM EDTA), photopolymer ELPEMER SD 2054, hydrophobic protective paint SD 2368 UV SG-DG (PETERS, Kempen, Germany) were used as received. Aqueous solutions were prepared with miliQ water (Simplicity^®^ MILLIPORE).

The base of the biosensors were chips covered with a layer of gold (Sens, The Netherlands).

### Appendix A.2. Apparatus and Chip Architecture

SPRI measurements were performed on a home-made apparatus consisting of a HeNe laser (JDS Uniphase, Edmund Industrial Optics, San Jose, CA, USA), two glass lenses L1 (f 3 mm) and L2 (f 300 mm), two polarizers (P1 and P2), a mirror, a prism and a CCD camera (QICam, QImaging, Vancouver, BC, Canada). NIH Image J version 1.42 software was used for evaluation of the SPRI images in 2D form.

The chip is an array of 108 active places. It has nine round measuring sites, each with 12 free gold surfaces. Using this chip, nine different solutions can be measured simultaneously without mixing the tested samples, and 12 separate SPRI measurements can be performed from a single 0.3 µL droplet of tested solution.

### Appendix A.3. Optimization of Concentration of Rabbit Polyclonal Antibody against HE 4

Optimization of the concentration of rabbit polyclonal antibody against HE 4 was performed at constant HE 4 concentration (114 pM). A chip preliminarily covered with cysteamine was treated with different concentrations of rabbit polyclonal antibody against HE 4 which has been previously activated with a mixture of EDC (250 nM) and NHS (250 nM) in carbonate buffer (pH 8.5). After 60 min of interaction the antibody was covalently immobilized on the chip, which was then rinsed with water and dried under a stream of argon. The first SPRi measurement was performed. The biosensor was treated with 114 pM solution of HE 4, rinsed with HBS-ES buffer and water, and finally dried under the stream of argon, after which the second SPRi measurement was performed. The analytical signal, which is the difference of the first and the second measurements, was plotted as a function of antibody concentration (Figure A1). On the basis of this graph, an antibody concentration of 20 ng mL^−1^ was selected as the optimal value for further experiments.
